# Editorial: Panic Buying: Human Psychology and Environmental Influence

**DOI:** 10.3389/fpubh.2021.694734

**Published:** 2021-05-19

**Authors:** S. M. Yasir Arafat, Sujita Kumar Kar, Russell Kabir

**Affiliations:** ^1^Department of Psychiatry, Enam Medical College and Hospital, Dhaka, Bangladesh; ^2^Department of Psychiatry, King George's Medical University, Lucknow, India; ^3^Faculty of Health, Education, School of Allied Health, Medicine, and Social Care, Anglia Ruskin University, Chelmsford, United Kingdom

**Keywords:** panic buying, buying psychology, consumer psychology, public health emergency, environmental stimuli, crisis

Panic buying (PB) is an emerging Research Topic. During this COVID-19 pandemic, it has got attention even though it has been noticed since a long time back during the crises (1, 2). Although there are several challenges newer studies have been coming out during this COVID-19 pandemic exploring several aspects of PB (1, 2). This Research Topic was aimed to highlight the different perspectives of panic buying comprehensively as much as possible which in turn can be used as a reference point for stakeholders. We targeted to discuss its historical perspectives, psychological explanations, sociological aspects, marketing dimensions, economics, supply chain management, industrial buying behavior, regional distributions and variation, disaster and emergency preparedness, the role of digital and social media, and preventive strategies. To our best knowledge, it is the first of its kind to approach organizing the possible thoughts on different perspectives of panic buying which would be useful for policymakers to prevent as well as manage panic buying incidents in future events if such an emergency arises.

As an issue, PB has got the recent attention of the academics and research community (1). It is defined as a “phenomenon of a sudden increase in buying of one or more essential goods in excess of regular need provoked by adversity, usually a disaster or an outbreak resulting in an imbalance between supply and demand” (3). Usually, it starts after an adverse environmental stimulus such as disaster, war, policy change; people buy necessary goods in excess amounts and creates a supply-demand imbalance (Arafat et al.; Arafat et al.). We proposed a complex interaction between several factors mentioned as primary, secondary, and tertiary factors (Arafat et al.) (Figure 1). As per our proposed *environmental stimuli and human psychology interaction* model adverse stimuli is the primary event to start PB behavior (Arafat et al.). One study replicated the concept that assessed PB in Bangladesh both in COVID-19 and non-COVID-19 context (Arafat et al.). The study revealed that the PB events in Bangladesh were precipitated by adverse environmental stimuli; identified that the people buy staples; and describe the implemented prevention strategies (Arafat et al.). Another study from China assessed the relationship of scarce consumption behavior with materialism and the need to belong during public health emergencies (Jin et al.). The study identified that the severity of the emergency event is positively associated with materialism, scarce consumption, and the need to belong even though the effect is transient (Jin et al.). This phenomenon is also explained by the *environmental stimuli and human psychology interaction* model. During this COVID-19 pandemic, adherent, and dysfunctional safety behaviors mediate the consumption pattern (Weismüller et al.). Mass media

**Figure 1:**
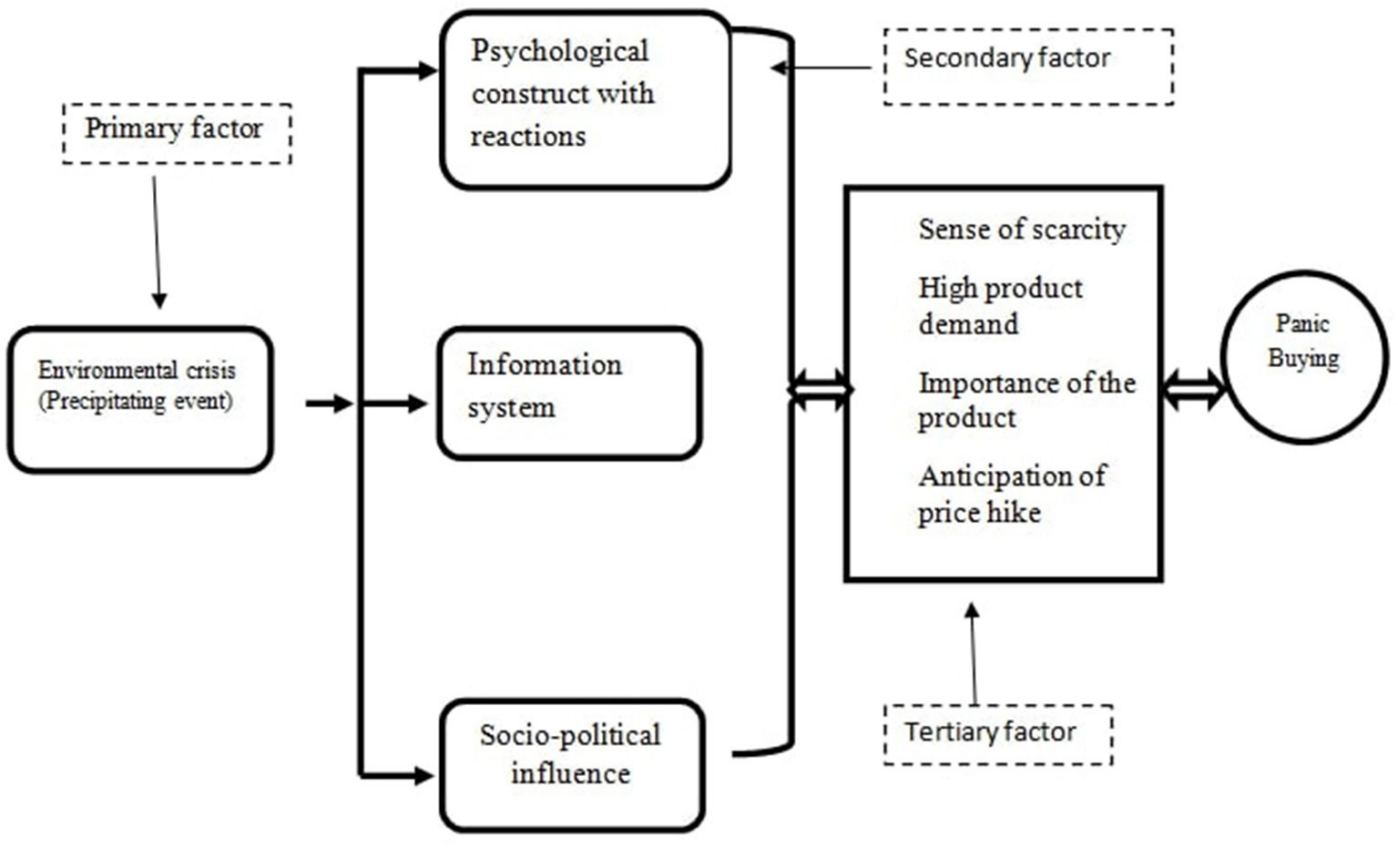
Environmental stimuli and human psychology interaction model [Adapted from (Arafat et al.)].

communication and long-term trust in the government affect safety behaviors during public health emergencies (Weismüller et al.). These factors also affect the PB behavior in several aspects and fit with the *environmental stimuli and human psychology interaction* model (Arafat et al.). It has also been explained from the perspective of certain behavioral and psychodynamic explanations (Cooper and Gordon). Supply restriction results in the generation of negative emotions in the consumers, which in turn result in PB behavior. Rajkumar, had attempted to explain the panic buying behavior from the perspective of the bio-psycho-social model. Biologically the panic buying behavior has a resemblance with the compulsive and hoarding phenomenon which might be due to shared neurobiological factors. Psychological explanation of panic buying might be explained on the basis of attachment to objects. The attachment becomes very crucial at the time of scarcity of the object. From the social perspective, panic buying can be understood. As per this, social exclusion and lack of social support are associated with excessive buying and stocking (Rajkumar). Group panic buying has also been reported during this COVID-19 pandemic. The propagation model to a greater extent explains the PB behavior (Fu et al.). The spread of negative information (both on online platforms and offline forums) evokes panic among people, compelling them to panic buying (Fu et al.). A study from the Iraqi Kurdistan region has revealed that lack of sensibility in social media posts may increase panic buying behavior (Arafat et al.). It has been seen that individuals who have more pandemic-related health fears and those who experience intense stress due to scarcity of products and restrictions in their availability are more likely to involve in panic buying (Georgiadou).

Still, there remains controversy that, whether panic buying should be considered as a diagnosable entity (i.e., pathology) or a normative behavior in its extreme at the face of stress (i.e., contextual phenomenon). This phenomenon is poorly studied in past; however; there is extensive research during this COVID-19 pandemic, to understand the phenomenon. The collection of this Research Topic still identifies that prevention of PB has got little attention despite being a common incidence during public health/environmental emergencies. More articles are coming out discussing the theoretical and phenomenological aspects whilst ignoring the prevention aspects. As PB is a closely related event of a public health emergency, prevention of PB should be considered as a regular package of emergency preparedness. Without the prevention of PB, the distressed people during public health emergencies wouldn't be benefitted. Then, all the scientific efforts would seem meaningless.

## Author Contributions

All authors listed have made a substantial, direct and intellectual contribution to the work, and approved it for publication.

## Conflict of Interest

The authors declare that the research was conducted in the absence of any commercial or financial relationships that could be construed as a potential conflict of interest.
